# Case Report: Complete response to four cycles of camrelizumab in a PD-L1 negative patient with advanced oral squamous cancer

**DOI:** 10.3389/fimmu.2025.1476455

**Published:** 2025-02-24

**Authors:** Jianqiao He, Guoning Yu, Yi Ma, Xiaoqiong Shi, Yingna Gao, Hongliang Zheng, Minhui Zhu, Caiyun Zhang

**Affiliations:** ^1^ Department of Otorhinolaryngology Head and Neck Surgery, The First Affiliated Hospital of Naval Medical University (Changhai Hospital of Shanghai), Shanghai, China; ^2^ The 909th Hospital, School of Medicine, Xiamen University, Zhangzhou, China

**Keywords:** immunotherapy, complete response, unresectable recurrent or metastatic squamus cell carcinoma, gene mutation, camrelizumab, case report

## Abstract

Immunotherapy has brought better survival benefits in the treatment of recurrent or metastatic head and neck squamous cell carcinoma (R/M HNSCC). However, owing to the lack of relevant biomarkers that could predict the efficacy of this treatment, it often has to be maintained. Here we report on a patient with stage IVA squamous cell carcinoma of the tongue who developed an unresectable lesion in the neck after surgery and radical chemoradiotherapy. After four cycles of intermittent immunotherapy with camrelizumab, complete response (CR) was achieved. Next-generation sequencing showed that the TP53/FANCA/FAT1 gene mutations and negative PD-L1 expression were involved. The patient has been followed up for 4 years without R/M. This situation has not been reported previously, suggesting that some patients can benefit from short-term immunotherapy and even achieve CR; moreover, there may be more molecular markers to be discovered that can predict the efficacy of immunotherapy. We can conduct in-depth research on relevant molecular markers, formulate personalized immunotherapy strategies and plans, and facilitate the development of new precision treatment strategies for HNSCC.

## Introduction

Oral cancer is one of the most common malignant tumors of head and neck squamous cell carcinoma (HNSCC). The incidence rate of tongue SCC among oral cancers is 25–40% (1). Postoperative radiotherapy combined with chemotherapy can achieve better local control and overall survival (OS) rates for locally advanced tongue SCC (2, 3), but 40% of patients still have recurrences (1). Some patients even develop unresectable R/M disease. R/M HNSCC has a poor prognosis, more than 50% of patients are unable to undergo salvage surgery (4), with a median OS of only 10–15 months (5).

Immune checkpoint inhibitors (ICIs)—compared with traditional chemotherapy, radiotherapy, and targeted therapy—have significantly improved the overall survival rate of some unresectable R/M HNSCCs. In the Updated Results of the Phase III KEYNOTE - 048 Study, the objective response rate of pembrolizumab monotherapy in patients with PD - L1 CPS ≥1 was 16.9% (6). Currently, PD-L1 is identified as a molecular marker that can predict the efficacy of immunotherapy; however, there are few others (7). Therefore, it is difficult to define the maintenance time of immunotherapy according to the current statement (8). The usual practice is to continue using the ICIs for 2 years or until clear progression or serious adverse reactions occur. In some patients who have achieved CR or sustained CR during early, midterm, or maintenance treatment, the medication time may be shortened or the ICIs can be used intermittently, thereby reducing the possibility of immune-related adverse events (irAEs) and unnecessary expense. Given this, future research should screen for stable and effective biomarkers for R/M HNSCC and conduct individualized treatment with short cycles or shortened medication times for some cases with early CR.

Here, we report for the first time a rare case of a patient with unresectable recurrent SCC of the tongue who was negative for PD-L1, had TP53/FANCA/FAT1 gene mutations, and achieved CR after short-term intermittent treatment with camrelizumab.

## Case presentation

**Figure 1:**
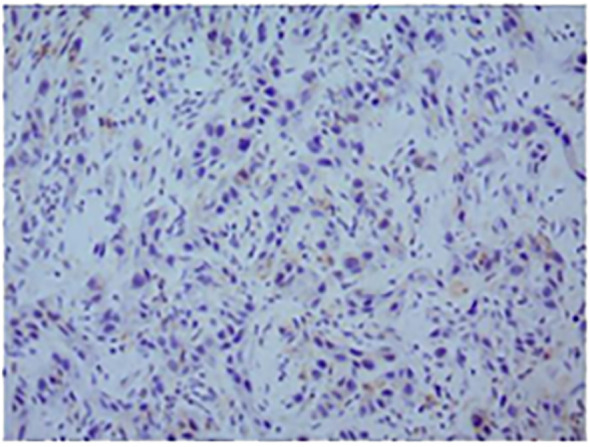
Representative micrographs of PD-L1 expression (original magnification, ×200). The positive rate of PD-L1 was less than 1%; the PD-L1 test result was negative.

In November 2016, a 55-year-old man visited the dental department of our hospital because of pain on the right tongue edge for more than 1 month. On November 14, 2016, right tongue resection, partial right mandibular resection, and right cervical lymph node dissection were implemented, along with left forearm flap transplantation for repair. Immunohistochemistry results were as follows: CK5/6 (+), P40 (+), P63 (+), Topo II (20%), Ki-67 (50%), P53 (-), and P16 (-). PD-L1 expression in tumor cells was negative (**Figure 1**). The patient was diagnosed with well-differentiated SCC of the tongue with right cervical lymph node metastasis, and the AJCC TNM stage was cT3N2bM0 IVA.

A total of six rounds of docetaxel 120 mg D1 and oxaliplatin 150 mg D1 (TP) regimen chemotherapy was started on May 24, 2017, during which postoperative radiotherapy was performed simultaneously. And the condition was evaluated as CR according to response evaluation criteria in solid tumors version 1.1 (RECIST v1.1) after treatment.

On May 19, 2019, an MRI scan of the neck showed that the left cervical lymph node was swollen with necrosis. The left neck mass was resected twice, on May 31and July 26, 2019, and the postoperative pathology showed SCC of the left neck.

**Figure 2:**
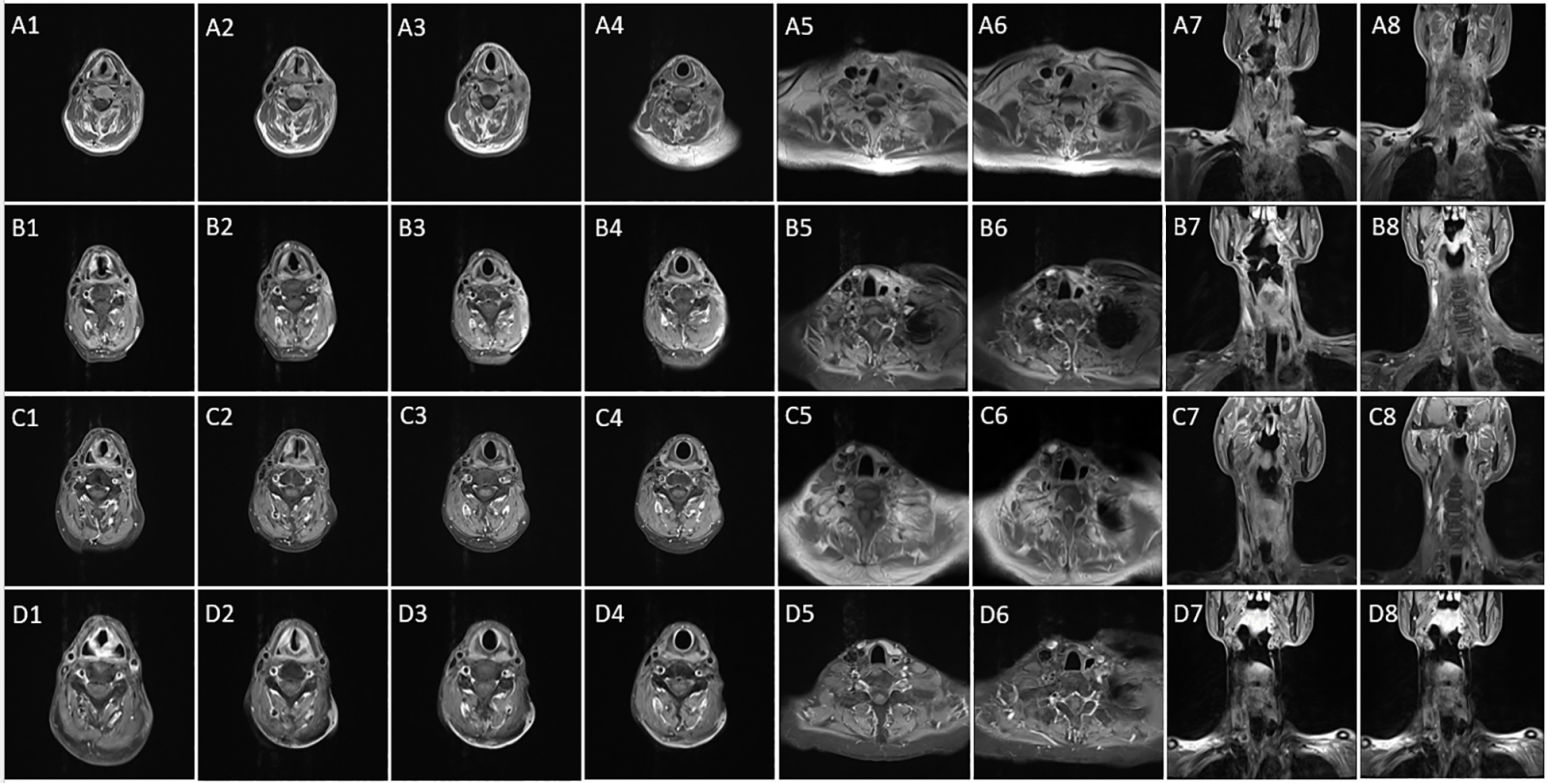
**(A1–8)** This tumor infiltrated the middle and upper neck as well as the base of the neck; the condition is now more advanced than before (November 24, 2019). **(B1–8)** The disease is close to complete response after four cycles of immunotherapy and two cycles of chemotherapy (April 3, 2020). **(C1–8)** According to RECIST v1.1 criteria, the patient’s disease is now in complete response (March 2, 2021). **(D1-8)** The disease remains in complete response (February 15, 2022).

On November 24, 2019, another MRI examination showed multiple enlarged and fused lymph nodes in the left side of the neck, some with necrosis, and obvious adhesions between some lymph nodes and the common carotid artery. This condition had progressed more than before (**Figure 2A1–8**), and it was assessed to be unresectable.

On November 26 and December 31, 2019, two cycles of paclitaxel 400 mg D1, cisplatin 140 mg D1, and Tegafur 40 mg/60 mg D1-14 (TPF), and camrelizumab 200 mg D1 were given. Due to measures to contain the COVID-19 epidemic in Shanghai in early 2020, he could not be readmitted to the hospital, so treatment was postponed.

On April 3, 2020, a neck MRI scan showed that the tumor had significantly improved compared with November 24, 2019 (**Figure 2B1–8**). He was then treated with camrelizumab 200 mg once in April and again in June 2020. Thereafter, he did not receive immunologic drugs or any other chemotherapy owing to economic constraints.

On June 15, 2020, another MRI scan showed that the lymph node metastasis in the left neck was smaller than before. No abnormally enlarged lymph nodes were found in the reexamination on March 2, 2021, and the treatment effect was evaluated as a CR (**Figure 2C1–8**). A follow-up MRI on February 15, 2022 showed continued remission (**Figure 2D1–8**).

**Figure 3:**
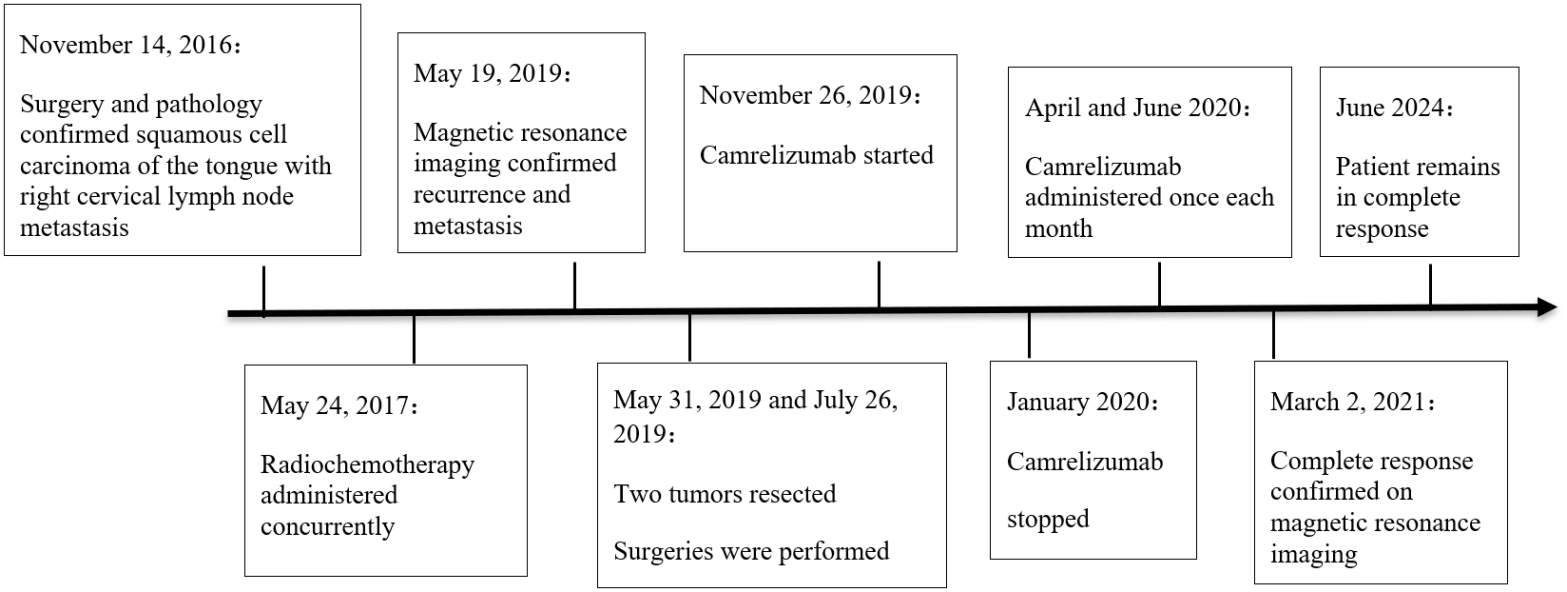
Timeline of the patient’s clinical course.

A timeline summarizing these events is shown in **Figure 3**. It has been more than 7 years since the patient first became ill. After treatment for recurrence, he has been regularly followed in our department for more than 4 years. He is in a state of continuous CR, and his current condition is stable. The patient’s Next-generation sequencing showed that there were TP53 gene nonsense mutation, FANCA gene frame shift mutation, FAT1 gene missense mutation and that TMB had 7.98 mutations/Mb.

## Discussion

Immunotherapy is currently recommended as the first-line treatment for R/M HNSCC. Compared with traditional regimens, it can significantly improve patient survival; however, only 20–30% of the patients benefit (9). There are also few molecular markers that can predict the efficacy of this modality and no standard for its maintenance time. The routine is to maintain it for 2 years or more until disease progression or intolerable toxicity. However, there are no literature reports on short-term medication or on shortening the medication time for individualized treatment according to the patient’s condition. The present article reports for the first time a case of CR achieved by short-term application of camrelizumab in a patient with unresectable locally recurrent HNSCC. The patient was followed up for 4 years without recurrence. He has negative PD-L1 expression and TP53/FANCA/FAT1 gene mutations, suggesting that in addition to PD-L1, these gene mutations may also be important molecular markers for immunotherapy. Short-term, intermittent application of immunotherapy in similar patients may enable them to achieve CR and obtain long-term survival benefits. In addition, large-sample studies can be conducted to verify relevant molecular markers and thus optimize individualized immunotherapy strategies and protocols. Thus, it may be possible to achieve the same therapeutic effects in R/M HNSCC patients while minimizing irAES and reducing the associated economic burden.

Molecular markers that could predict the efficacy of immunotherapy are currently a research hotspot in the context of precision medicine. In phase III of the KEYNOTE-048 clinical study, the biomarker PD-L1 stratified design was used to optimize the selection of immunotherapy benefit groups. As a result, a significant OS benefit was observed in the patient group with a PD-L1 combined positive score equal to or greater than 20. Furthermore, the 5-year survival rate in such patients ranged from approximately 15% to 20%. Therefore, PD-L1 is currently an effective molecular marker for HNSCC. TP53 is the most common somatic gene mutation in HNSCC. Once TP53 gene mutated, it will transform from tumor suppressor gene to oncogene. HSNCC patients with the TP53 mutation respond poorly to immunotherapy and chemotherapy (10), as related to the generally poor prognosis of HNSCC. However, a previous study found that HNSCC patients carrying TP53/FAT1 commutations had higher PD-L1 expression levels and longer median survivals than those carrying only TP53 gene mutations (11). Currently, the regulatory mechanism of the FAT1 gene is not completely clear; it is considered to be a tumor suppressor or driver (12). The FAT1 gene mutation is the second most common mutation in HNSCC. It is an important factor in the occurrence and development of HNSCC and an independent predictor of a poor prognosis (13, 14). However, combined with HPV (-) in HNSCC patients, the FAT1 gene mutation is significantly associated with better OS (15). The FANCA gene is a DNA damage repair gene. FANCA gene mutations can change the pathways of cellular energy metabolism, as well as alter DNA stability and lead to mitochondrial functional damage, thereby aggravating the accumulation of DNA damage in HNSCC cell models and exacerbating the disease (16). There is currently no clinical application research related to immunotherapy for HNSCC.

In the face of multiple genetic mutations and potential heterogeneity in HNSCC patients, it seems unrealistic to screen for a single molecular marker to predict the effect of immunotherapy. Multimodal combinations of molecular markers represent opportunities and challenges for predicting the benefits of ICIs and need to be further explored. Our patient with negative PD-L1 expression achieved an astonishing CR with short-term, intermittent immunotherapy, suggesting that his sensitivity to immunotherapy may be related to other molecular markers. Thus, it is important to further explore other potential predictive biomarkers. TP53, FAT1, and FANCA gene mutations revealed by next-generation sequencing suggest that this patient’s sensitivity to immunotherapy may stem mainly from mutations in these genes. Currently, we have not seen any reports of patients with negative PD-L1 expression and simultaneous mutations in TP53, FAT1, and FANCA genes who are sensitive to immunotherapy. Therefore, our findings may provide new directions for joint research on immunotherapy-related molecular markers.

In current clinical practice for some patients with unresectable local R/M HNSCC, some domestic and foreign experts recommend continued ICI treatment for these patients when they achieve clinical CR with no serious adverse reactions. It is often difficult for head and neck oncologists as well as patients to choose the discontinuation points for ICIs. This is an important issue that, in the current era of precision treatment of head and neck tumors, needs to be solved urgently. The discovery of relevant molecular markers, especially those with TP53/FANCA/FAT1 gene mutations, in this case report may suggests that short-term, intermittent immunotherapy may achieve long-term survival benefits in some patients with R/M HNSCC, and make it possible to personalize immunotherapy while also minimizing irAES and reducing the patients’ economic burden. We reviewed the literature and found that there were no other reports of such cases. Our results may provide new clues for the study of molecular markers in immunotherapy.

## Data Availability

The original contributions presented in the study are included in the article/supplementary materials, further inquiries can be directed to the corresponding author/s.
